# The Caregiver Support Model for Informal Caregivers of Frail Older Adults: Randomized Controlled Trial

**DOI:** 10.2196/71638

**Published:** 2025-11-03

**Authors:** Cyrus Lap Kwan Leung, Kin-Kit Li, Dannii Y Yeung, Alice Ming Lin Chong, Marcus Yu Lung Chiu, Xin Guan, Tit Wing Lo

**Affiliations:** 1 The Jockey Club School of Public Health and Primary Care, Faculty of Medicine, Chinese University of Hong Kong, Hong Kong, China (Hong Kong); 2 Department of Social and Behavioural Sciences, College of Liberal Arts and Social Sciences, City University of Hong Kong, Tat Chee Avenue, Kowloon Tong, Hong Kong, 999077, China (Hong Kong), 852 34428119; 3 Felizberta Lo Padilla Tong School of Social Sciences, Saint Francis University, Hong Kong, China (Hong Kong); 4 Caregiving Research and Development Centre, Saint Francis University, Hong Kong, China (Hong Kong); 5 Department of Social Work and Social Administration, Faculty of Social Sciences, University of Hong Kong, Hong Kong, China (Hong Kong)

**Keywords:** informal caregiving, caregiver support, randomized controlled trial, caregiving resources, caregiving burden

## Abstract

**Background:**

Caregivers of frail older adults face substantial challenges, often managing their own health while providing care. To address these issues, we developed the caregiver support model (CSM), a structured approach that uses systematic assessment, personalized intervention planning, and sustained support to address informal family caregivers’ diverse and evolving needs and leverage their resources.

**Objective:**

This study aims to evaluate the effectiveness of CSM.

**Methods:**

A blinded cluster randomized controlled trial was conducted across 8 centers providing services for older adults in Hong Kong. The CSM is a social worker–guided intervention that integrates a structured assessment of caregiver needs and resources, personalized service planning, and ongoing monitoring over 6 months. Meanwhile, the control group continued with their usual procedures without a standardized caregiver assessment. Data were collected at baseline, 3 months, and 6 months.

**Results:**

We recruited 565 informal family caregivers (281/565, 49.7% CSM intervention; 284/565, 50.3% standard care control). Both groups improved over time; compared with the control group, the CSM produced greater reductions in caregiver needs, particularly in role conflict, and greater gains in resources, such as health awareness. Improvements were more pronounced at 6 months compared to 3 months, indicating a lasting effect and consolidation of gains. The intervention was particularly effective for caregivers in other relationships (not spouse or child) and those with higher education than spousal caregivers.

**Conclusions:**

These findings highlight the importance of long-term tailored interventions that adapt to the evolving needs of caregivers through systematic assessment. The CSM offers a promising approach to enhancing the well-being of caregivers and managing the complex demands of caregiving, particularly in an aging population.

## Introduction

### Growing Care Demands and Service Gaps

Hong Kong, like many other high-income international cities, is increasingly grappling with the complex challenges posed by an aging population. The number of adults aged ≥60 years surged from 1.44 million (20.1% of the total population) in 2012 to approximately 2.2 million (29.9%) in 2022, with projections indicating a further rise to 3.21 million (40.1%) by 2050 [1]. This demographic shift has been accompanied by a growing reliance on informal caregivers. In 2021, over three-quarters of older adults needing long-term care (16.6% of those aged ≥60 years) relied on informal caregiving [2]. Given that long-term care needs often stem from age-related frailty, which is associated with increased dependency in mobility, personal care, and supervision, caregivers are frequently tasked with managing complex physical, emotional, and logistical demands, especially in smaller households where responsibilities often fall on the spouse or adult child [3-5]. These converging trends underscore the pressing need for a well-structured support system that both acknowledges the critical role of informal caregivers and provides resources to enhance their ability to sustain the well-being and dignity of Hong Kong’s aging population.

The public health and social welfare systems in Hong Kong currently face significant gaps in providing adequate support for family caregivers, driving many to rely on private care services [6]. While these private services provide essential assistance, they are often expensive for low-income families [4,5]. To bridge this gap, the Social Welfare Department has introduced a range of services to support caregivers [7], including a designated caregiver support hotline, an information portal, and community-based programs for older adults and their caregivers. In addition, local nongovernmental organizations (NGOs) offer various services, such as information dissemination, referrals, skills training, and support groups. Despite these efforts, recent findings highlight persistent structural limitations, such as inadequate workplace flexibility, fragmented service access, and a lack of cross-sector coordination [8,9]. In addition, a critical shortfall remains in the form of unstandardized guidelines for assessing and addressing caregiver needs [10], along with limited training and preparedness among frontline social workers. This structural deficiency hampers the effective management and distribution of resources. To address these challenges, we developed the caregiver support model (CSM), which offers a structured and comprehensive framework for assessment, case management, and training. The CSM is a critical step toward a more integrated approach to supporting caregivers, helping to alleviate the growing strain on informal family caregivers within Hong Kong’s health care system. Endorsing an assessment-based, balanced, caregiver-centered, and case-management approach, the CSM has the potential to inform future practices in other countries or cities with similar social services infrastructure.

### Dynamic Nature of Caregiving and Theoretical Foundations of the CSM

The CSM is grounded on well-established theoretical frameworks, incorporating key elements from the stress process model [11], the stress and coping model [12,13], and empowerment and strengths-based approaches. The stress process model highlights how primary stressors (eg, functional limitations of the care recipient) and secondary strains (eg, role conflict and psychological demands) shape caregiver burden, while coping strategies and available resources act as mediators. The stress and coping model complements this by emphasizing the process of stress appraisal, coping, and reappraisal over time, illustrating why structured assessment and repeated follow-up are necessary. Together, these models conceptualize caregiving as an evolving process and provide the rationale for interventions that both reduce stressors and strengthen protective resources. Central to the CSM is the empowerment of caregivers, which encourages them to recognize their strengths, advocate for their needs, and adopt sustainable caregiving practices. This is closely aligned with the strengths-based approach [14,15], which focuses on identifying and harnessing caregivers’ inherent capacities to overcome challenges, thereby fostering resilience and better adaptation to the caregiving role.

Recognizing caregiving as a dynamic process, the CSM identifies distinct stages of caregiving [16], each presenting unique challenges and needs. Accurately assessing these needs is crucial as they shift alongside the care recipient’s illness progression and the evolving role and responsibilities of the caregiver [17,18]. In Hong Kong, several standardized tools are used for caregiver assessment, such as the 12-item Zarit Burden Interview (ZBI-12) [19], the Perceived Stress Scale [20], and the 21-item Depression Anxiety Stress Scale [21]. While these tools offer valuable insights, they often fail to capture the multifaceted nature of caregiving, particularly in areas such as role conflict and the personal needs of caregivers, both of which significantly impact caregiver burden and satisfaction [22-24]. This limitation highlights the need for a comprehensive, standardized tool tailored for caregivers of frail older persons. Such a tool must not only capture caregivers’ diverse needs but also evaluate the resources that help them cope with caregiving demands. Needs include physiological, psychological, social, and role-related challenges, while resources encompass personal, relational, and community assets that buffer stress [25,26]. To address this gap, the Caregiver Needs and Resources Assessment (CNRA) [10] was developed as a holistic measure. In the CNRA, needs are assessed through subscales covering physiological strain, psychological distress, social support needs, role conflict, and care recipient needs. Resources are defined to include both personal assets (eg, self-efficacy, spirituality, and health awareness) and external supports (eg, family support, closeness with the care recipient, community resources, and responsibility). This dual focus allows the CNRA to guide interventions that both reduce unmet needs and strengthen supports.

Another key component of the CSM is case management, which is essential for coordinating services and interventions that are tailored to the specific needs and circumstances of caregivers [27,28]. In Hong Kong, however, the lack of standardized processes for assessing caregiver needs often leads to subjective evaluations by social workers or health care professionals. To address this issue, the CSM applies the CNRA. This tool empowers social workers to develop client-centered intervention plans and allocate resources more effectively, ensuring that the support provided is closely aligned with each caregiver’s unique situation and needs in performing their caregiving role.

This paper aims to evaluate the effectiveness of the CSM through a clustered randomized controlled trial. The CSM represents a significant innovation in gerontology and social work, offering social workers a scientifically validated, comprehensive, and caregiver-centered framework to assist and empower family caregivers of frail older adults. The model includes a range of tools, such as the CNRA, an online caregiver management system, personalized intervention and monitoring templates, and detailed operational guidelines. By rigorously evaluating the CSM’s impact on caregiver support, this study seeks to advance the development of evidence-based strategies in informal care, addressing the complex needs of caregivers of frail older adults in Hong Kong and across the world. We hypothesized that participants in the CSM group would have more favorable outcomes compared with those in the control group at the end of the intervention trial.

## Methods

### Study Design

The study used a cluster randomized controlled trial design, with parallel allocation to either intervention or control arms, to evaluate the effectiveness of the CSM. The CSM is a social worker–guided intervention and comprises four key components: (1) the CNRA [10], (2) the formulation of a caregiver intervention plan, (3) service provision and monitoring, and (4) evaluation and termination. Eight centers from the 4 participating NGOs were randomly assigned to either the intervention or control group. Although the service administrators could not be blinded from the intervention, participants of the study were not aware of their assigned conditions. All caregiver participants, regardless of their conditions, completed the CNRA before their first meeting with the social worker. The intervention group followed the CSM protocol, in which the CNRA scores were shared with the social workers, who explained the need and resource scores to the participants and then generated a personalized intervention plan for each of them, followed by recommending appropriate services. The social workers in the control group, without knowledge of the CNRA scores, adhered to their own standard procedures in assessing and differentiating caregivers’ needs when they sought assistance. The intervention was delivered between January 1, 2022, and August 31, 2022, with data collection at baseline, 3 months, and 6 months after intervention, concluding by February 28, 2023. The fifth wave of the COVID-19 pandemic led to a temporary pause in recruitment, prompting adaptations in service delivery and subsequent division of the recruitment period into 2 phases (phase 1: January 1, 2022, to April 30, 2022; phase 2: May 1, 2022, to August 31, 2022). The study design and reporting followed the CONSORT-EHEALTH (Consolidated Standards of Reporting Trials of Electronic and Mobile Health Applications and Online Telehealth) guidelines (Checklist 1) to ensure transparency and methodological rigor in the conduct and reporting of this randomized controlled trial.

### Participants

Participants in the study were informal family caregivers of frail older adults recruited offline from 8 centers of the 4 participating NGOs in Hong Kong. Eligibility criteria included being an unpaid primary caregiver of a family member aged ≥60 years, being at least the age of 21 years, either newly assuming caregiving responsibilities or facing an urgent need for assistance, scoring ≥13 on the Short-Form ZBI-12 to indicate their needs for receiving service support, as well as with the care recipients scoring ≥3 on at least 1 item of the activities of daily living or instrumental activities of daily living. Exclusion criteria included having received formal psychological intervention within the past 3 months. A total of 580 eligible caregivers were initially recruited, with 565 completing the CNRA at baseline (intervention group: 281/565, 49.7%; control group: 284/565, 50.3%). A total of 66 caregivers did not complete all of the follow-up assessments, resulting in an 11.7% (66/565) dropout rate.

### Intervention Protocol

The intervention protocol of the CSM involved a structured process that used the CNRA to assess caregivers’ needs and available resources. Once eligible caregivers were identified, a research assistant or a program worker administered the CNRA (T1), and the results were uploaded to an online management system for review by the intervention group’s social workers. These social workers then met with the caregivers to discuss their CNRA results and develop intervention plans, using spider web charts to visually illustrate needs and resources, thereby enhancing caregivers’ awareness of their own strengths and challenges. To empower caregivers in managing the services they receive, they were encouraged to prioritize their needs based on the discussion surrounding the test scores. The support services received by the caregivers in the intervention group were customized based on their needs and resources, while the participants in the control group received regular support services as usual.

Due to the COVID-19 pandemic, adaptations were made, including extended timelines and the use of mixed methods (face-to-face, video calls, and Zoom [Zoom Communications, Inc] meetings) for the first meeting. Regular follow-ups, either meetings, phone calls, or SMS text messaging, were conducted every month, with each meeting lasting no less than 15 minutes. There was also a midterm evaluation at 3 months (T2) after the baseline assessment, in which the caregivers’ needs and resources were assessed again using the CNRA. The intervention plans were then adjusted based on the updated CNRA results. The final CNRA assessment took place 6 months after intake (T3), determining whether the intervention would be continued or terminated. In contrast, social workers in the control group, who were unaware of the CNRA scores, relied on personal judgment and observations to guide their intervention plans. The same CNRA assessment schedule at T2 and T3 was adopted for the control group, even though the scores were not disclosed to the social workers in this group.

The development of the CSM was guided by the stress process model [11] and the stress and coping model [12,13]. With reference to the stress process model, the CNRA was structured to capture primary stressors (eg, care recipient’s needs), secondary strains (role conflict, psychological needs, and social support needs), and mediating resources (self-efficacy, family support, spirituality, community resources, and other supportive assets). The stress and coping model informed the intervention process: the baseline CNRA and spider chart feedback facilitated caregivers’ appraisal of needs and resources; the individualized service plan supported both problem-focused coping (eg, training and resource referral) and emotion-focused coping (eg, counseling and peer support); and the 3- and 6-month reassessments enabled structured reappraisal and adjustment. Together, these frameworks shaped the CSM as a structured process to assess needs, mobilize resources, and reinforce coping over time. A detailed CSM protocol is provided in Multimedia Appendix 1. This study was registered at ClinicalTrials.gov (identifier: NCT04272918). We adhered to the registered protocol (version as of February 21, 2020) except for the changes in some of the outcome measures (ZBI-12 and World Health Organization-5 were replaced by CNRA and Positive Aspects of Caregiving Scale [PACS]) after considering the input from participating NGOs. Quality assurance measures included recording and reviewing the first and second meetings with caregivers using a standardized checklist to ensure adherence to the protocol. Adherence rates for both meetings were assessed across centers, with research assistants providing timely feedback to social workers for continuous improvement.

### Evaluation and Measurements

The following measurement tools were used in the offline baselines and follow-up assessments for assessing the primary outcomes (that are direct and immediate) and the secondary outcomes (that are more general and distal).

#### Primary Outcomes

##### CNRA Tool

The CNRA [10] was used to conduct a comprehensive assessment of the needs and resources of the participating caregivers. A revised version of the original 36-item tool was adopted (Leung et al, unpublished data), incorporating minor wording updates to the closeness with the care recipients subscale. CNRA assesses 5 caregiver needs domains (physiological, role conflict, care recipient’s needs, psychological, and social) and 7 resource domains (spirituality, self-efficacy, responsibility, community resources, family support, closeness with the care recipient, and healthy lifestyle). Participants rated each item on a 5-point Likert scale from 1=never to 5=all the time about their caregiving experience in the past month. Higher scores on resource subscales reflect more perceived resources; higher scores on need subscales reflect greater unmet needs. Sample items include “Caring for him/her keeps me from getting enough sleep” (needs) and “Family members help me manage the difficulties of caregiving” (resources). The CNRA demonstrated high internal reliability, with the Cronbach α ranging from 0.74 to 0.97 across different subscales and time points. Two overall need scores are presented: one using the full scale and the other excluding the physiological and psychological needs (designated as “best practice”). This distinction was made because physiological needs are typically addressed through medical interventions, while psychological needs can generally be improved by social workers’ active interventions that benefit both intervention and control groups.

##### PACS Measure

The 11-item PACS was used to measure the positive feelings associated with caregiving, which consisted of the self-affirmation (affirming aspects) and outlook on life (enriching aspects) subscales. It uses a 5-point Likert scale from 1=not at all to 5=all the time, with higher scores indicating greater caregiving satisfaction. Sample items include “Given more meaning to my life” and “Enabled me to learn new skills.” The PACS has demonstrated high test-retest reliability and moderate construct validity, with the Cronbach α ranging from 0.82 to 0.89 at 3 time points.

### Secondary Outcomes

#### EQ-5D-5L

The EQ-5D-5L [29] was used to assess the quality of life and well-being across 5 dimensions: mobility, self-care, usual activities, pain or discomfort, and anxiety or depression. It includes a visual analog scale and 5 questions rated on a 5-point Likert scale, with higher scores indicating better health and well-being. The EQ-5D-5L has been validated for use in Hong Kong [30], with Cronbach α ranging from 0.70 to 0.75 in this study.

#### 12-item General Health Questionnaire

The 12-item General Health Questionnaire (GHQ-12) [31] was used to assess the participants’ mental health status. Respondents rated 12 statements using a 4-point Likert scale from 0=not at all to 3=all the time, with higher scores reflecting greater psychiatric distress. The GHQ-12 has been validated for the Chinese population and has demonstrated good construct validity and predictive reliability [32], with Cronbach α ranging from 0.86 to 0.88 in this study.

#### Peace of Mind Scale

The Peace of Mind Scale (PoMS) [33] was used to assess affective well-being. Participants rated 7 items on a 5-point Likert scale from 1=not at all to 5=all the time, with higher scores indicating greater peace of mind. Sample items include “My mind is free and at ease.” The PoMS demonstrated good reliability, with Cronbach α ranging from 0.92 to 0.94 in this study.

### Statistical Analysis

The demographic characteristics of caregivers and their care recipients in both intervention and control groups were summarized using mean scores and SDs for continuous variables, and percentages for categorical variables. Baseline equivalence between the groups was assessed using 2-tailed *t* tests for continuous variables and Fisher exact test for categorical variables. The 2-tailed *t* tests were also used to compare primary and secondary outcome measures between the intervention and control groups at baseline. The effectiveness of the intervention was evaluated using an intention-to-treat approach, which included all participants according to their original group assignment, with missing data imputed via chained equations [34].

To examine intervention effects, we modeled change scores (T2–T1 and T3–T1) as the primary outcomes. Multilevel regression models were estimated with random intercepts for centers to account for the clustered trial design. Models adjusted for the demographic characteristics of both caregivers and care recipients, including the caregiver’s age, sex, education level, long-term illness, marital status, years of caregiving, and caregiving stage, as well as the care recipient’s age, sex, and functional status (activities of daily living and instrumental activities of daily living). Robust small-sample corrections (cluster-robust variance estimators, specifically the bias-reduced linearization adjustment) were applied to SEs and *P* values to improve the reliability of inference given the limited number of clusters (in the center level). The potential moderating effects of caregiver and care recipient characteristics on the primary outcomes were assessed using interaction terms between the intervention condition and each demographic variable (eg, education and caregiver-recipient relationship) in separate multilevel regression models. Each model included the intervention, the moderator, their interaction, and a consistent set of covariates. All models accounted for clustering within centers and were estimated using imputed datasets.

### Ethical Considerations

The Human Subject Ethics Subcommittee of the City University of Hong Kong granted ethical approval for the study (reference 3-7-201905-01). Informed consent was obtained from all participants offline, ensuring voluntary participation. Personal identifiers were removed from the dataset, and all data were accessible only to authorized research personnel. Participants were compensated for their time involvement with an HK $150 (US $19.30) supermarket cash voucher upon completing the assessment survey of each round (T1, T2, and T3).

## Results

### Demographic Characteristics and Baseline Assessment of the Participants

**Figure 1.**
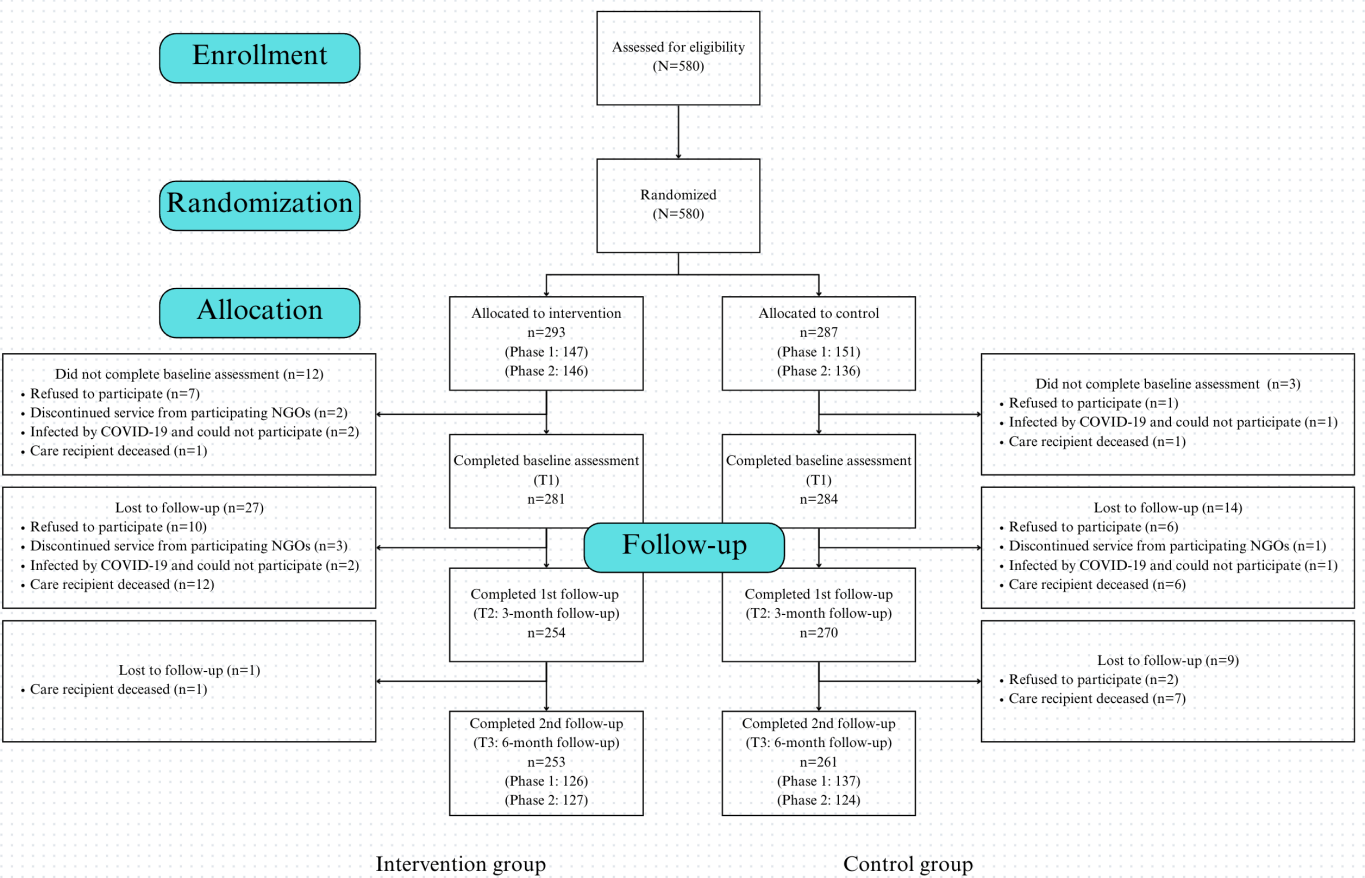
CONSORT-EHEALTH (Consolidated Standards of Reporting Trials of Electronic and Mobile Health Applications and Online Telehealth) flow diagram of the 2 study groups. NGO: nongovernmental organization.

Participant enrollment and allocation are shown in Figure 1. The study sample consisted of caregivers with an average age of 67.0 (SD 11.7) years, the majority of whom were female (478/565, 85%). Educational attainment varied, with the largest group having completed secondary school (234/565, 41%). Most caregivers were married (424/565, 75%), and a significant proportion (439/565, 78%) reported having a chronic disease. The care recipients had an average age of 81.2 (SD 8.9) years, with a majority being male (333/565, 59%). Participant characteristics for the entire sample and by study site information are presented in Table 1 and Table S1 of Multimedia Appendix 1, respectively. No important harms or unintended effects were found in each group.

**Table 1.** Descriptive statistics of the participants (N=565)^a^.

	Overall			Intervention (n=281)			Control (n=284)			*P* value
Characteristics of the caregivers										
Age (y), mean (SD; range)	67.0 (11.7; 21 to 96)			66.7 (12.3; 21 to 96)			67.3 (11.1; 26 to 88)			.52
Sex, n (%)										.27
Male	87 (15.4)			48 (17.1)			39 (13.7)			
Female	478 (84.6)			233 (82.9)			245 (86.3)			
Education level, n (%)										.58
No education received	35 (6.2)			19 (6.8)			16 (5.6)			
Private school	20 (43.5)			11 (3.9)			9 (3.2)			
Primary school	174 (30.8)			94 (33.5)			80 (28.2)			
Secondary school	234 (41.4)			113 (40.2)			121 (42.6)			
Certificate or diploma	35 (6.2)			16 (5.7)			19 (6.7)			
Undergraduate or greater	67 (11.9)			28 (10.0)			39 (13.7)			
Marital status, n (%)										.68
Married	424 (75.0)			213 (75.8)			211 (74.3)			
Not married	141 (25.0)			68 (24.2)			73 (25.7)			
Long-term illness, n (%)										.32
With	439 (77.7)			215 (76.5)			224 (78.9)			
Without	126 (22.3)			66 (23.5)			60 (21.1)			
Caregiving stage, n (%)										.29
Stage 1	40 (7.1)			16 (5.7)			24 (8.5)			
Stage 2	66 (11.7)			32 (11.4)			34 (12.0)			
Stage 3	119 (21.1)			59 (21.0)			60 (21.1)			
Stage 4A	126 (22.3)			75 (26.7)			52 (18.3)			
Stage 4B	201 (35.6)			95 (33.8)			106 (37.3)			
Stage 5	12 (2.1)			4 (1.4)			8 (2.8)			
Characteristics of the care recipients										
Age (y), mean (SD; range)	81.2 (8.9; 61 to 106)			80.9 (8.8; 61 to 106)			81.5 (9.0; 61 to 102)			.43
Sex, n (%)										.36
Male	333 (58.9)			171 (60.9)			162 (57)			
Female	232 (41.1)			110 (39.1)			122 (43)			
IADL^b^, mean (SD; range)	8.1 (4.4; –3.2 to 18.0)			2.0 (0.6; 0.0 to 18.0)			1.7 (0.5; –3.2 to 18.0)			<.001
ADL^c^, mean (SD; range)	4.1 (4.2; –6.2 to 18.0)			2.2 (0.7; 0.0 to 18.0)			2.0 (0.5; –6.2 to 18.0)			<.001

a Imputed data are used.

b IADL: instrumental activities of daily living.

c ADL: activities of daily living.

At baseline, the intervention group reported a significantly higher overall need score (best practice) compared to the control group (*t*_563_=3.20; *P*=.001; mean difference [MD] 0.2, 95% CI 0.1-0.4). In terms of needs subscales, the intervention group scored significantly higher in role conflict (*t*_563_=2.96; *P*=.003; MD 0.2, 95% CI 0.1-0.4), and care recipient need than the control group at the baseline (*t*_563_=3.20; *P*=.001; MD 0.3, 95% CI 0.1‐0.4). No significant differences were observed in physiological needs (*t*_563_=1.91; *P*=.06; MD 0.2, 95% CI −0.0 to 0.3), psychological needs (*t*_563_=1.64; *P*=.10; MD 0.2, 95% CI −0.0 to 0.3), and social support needs (*t*_563_=1.73; *P*=.08; MD 0.2, 95% CI −0.0 to 0.4), indicating baseline equivalence between the groups in these domains.

Regarding resources, the intervention group had a lower overall mean score at baseline compared to the control group (*t*_563_=−2.49; *P*=.01; MD −0.1, 95% CI −0.2 to −0.0). Subscales such as spirituality (*t*_563_=−2.26; *P*=.02; MD −0.2, 95% CI −0.4 to −0.0), family support (*t*_563_=−2.53; *P*=.01; MD −0.2, 95% CI −0.4 to −0.0), and closeness with the care recipient (*t*_563_=−2.09; *P*=.04; MD −0.2, 95% CI −0.3 to −0.0) also showed significant differences between the 2 groups at baseline.

For secondary outcomes, no significant differences were found in GHQ-12 (*t*_563_=1.06; *P*=.29; MD 0.0, 95% CI −0.0 to 0.1) and peace of mind (*t*_563_=−1.00; *P*=.32; MD −0.1, 95% CI −0.2 to 0.1), further indicating baseline equivalence. There were no significant differences in the affirming (*t*_563_=1.67; *P*=.10; MD 0.1, 95% CI −0.0 to 0.3) and enriching *(t*_563_=−1.64; *P*=.10; MD −0.1, 95% CI −0.3 to 0.0) subscales of the PACS between the 2 groups at baseline. However, the intervention group exhibited a significantly lower EQ-5D score compared to the control group (*t*_563_=−2.79; *P*=.005; MD 0.0, 95% CI −0.1 to −0.0). Details of baseline equivalence are shown in Table 2.

**Table 2.** Baseline comparison on outcome measure between the intervention and control groups (N=565)^a^. The 12-item General Health Questionnaire scores in the range of 0‐36 can be obtained by multiplying the GHQ-12 mean score by 12.

	Overall, mean (SD; range)			Intervention (n=281), mean (SD)		Control (n=284), mean (SD)		*P* value
Primary outcomes								
Needs (full scale)	2.6 (0.9; 1 to 5)			2.7 (0.9)		2.5 (0.9)		.005^b^
Physiological need	2.4 (1.0; 1 to 5)			2.5 (1.0)		2.3 (1.0)		.06
Role conflict	2.3 (1.0; 1-5)			2.4 (1.0)		2.2 (0.9)		.003^b^
Care recipient need	2.9 (1.0; 1-5)			3.0 (1.0)		2.8 (1.0)		.001^b^
Psychological need	2.7 (1.1; 1 to 5)			2.8 (1.1)		2.7 (1.1)		.10
Social support need	2.8 (1.3; 1 to 5)			2.9 (1.3)		2.7 (1.3)		.08
Needs (best practice)	2.7 (0.9; 1 to 5)			2.8 (0.9)		2.6 (0.9)		.001^b^
Resources	3.3 (0.6; 1 to 5)			3.2 (0.6)		3.4 (0.6)		.01^c^
Spirituality	3.4 (1.1; 1 to 5)			3.3 (1.1)		3.5 (1.0)		.02^c^
Self-efficacy	3.3 (0.8; 1 to 5)			3.3 (0.8)		3.3 (0.8)		.56
Responsibility	4.1 (0.8; 1 to 5)			4.1 (0.7)		4.1 (0.8)		.80
Community resources	2.5 (1.2; 1 to 5)			2.5 (1.2)		2.6 (1.2)		.16
Family support	3.3 (1.0; 1 to 5)			3.2 (1.1)		3.4 (1.0)		.01^c^
Closeness with care recipient	3.4 (0.9; 1 to 5)			3.4 (0.9)		3.5 (0.9)		.04^c^
Health awareness	2.8 (1.1; 1 to 5)			2.8 (1.1)		2.9 (1.0)		.29
PACS^d^	3.2 (0.8; 1 to 5)			3.3 (0.8)		3.2(0.8)		.88
Affirming	3.3 (0.8; 1 to 5)			3.4 (0.8)		3.3 (0.8)		.10
Enriching	3.1 (0.9; 1 to 5)			3.1 (0.9)		3.2 (0.8)		.10
Secondary outcomes								
GHQ-12 (mean score)	1.2 (0.5; 0 to 3)			1.3 (0.5)		1.2 (0.5)		.29
Peace of Mind Scale	3.1 (0.9; 0 to 3)			3.1 (0.9)		3.2 (0.9)		.32
EQ-5D	0.8 (0.2; −0.5 to 1)			0.7 (0.2)		0.8 (0.2)		.005^b^

a Imputed data are used.

b *P*<.01.

c *P*<.05.

d PACS: Positive Aspects of Caregiving Scale.

### Multilevel Regressions Examining Intervention Effects

Intraclass correlation coefficients (ICC) for the center-level random effects ranged from 0.00 to 0.11 across outcomes (Table 3), indicating that only a small proportion of variance was attributable to clustering. This supports the use of random intercepts for centers to reflect the clustered trial design, while showing that clustering effects were relatively modest.

**Table 3.** Multilevel regressions predicting changes in the caregiving needs and resources (N=565)^a^.

	T2-T1								T3-T1							
	Control (n=284)			Intervention (n=281)			ICC^b^	*P* value	Control (n=284)			Intervention (n=281)			ICC	*P* value
	Estimated mean (95% CI)		Missing, n	Estimated mean (95% CI)		Missing, n			Estimated mean (95% CI)		Missing, n	Estimated mean (95% CI)		Missing, n		
Primary outcomes																
Needs (full scale)	*−0.26* *(−0.39 to −0.13)* ^c^		15	*−0.31* *(−0.47 to −0.14)*		27	0.01	.60	*−0.21* *(−0.42 to −0.00)*		24	*−0.30* *(−0.52 to −0.07*		28	0.00	.10
Physiological need	*−0.13* *(−0.27 to −0.00)*		15	−0.12 (−0.29 to 0.06)		27	0.00	.75	0.02 (−0.28 to 0.32*)*		24	0.04 (−0.27 to 0.35)		28	0.004	.84
Role conflict	−0.24 (−0.56 to 0.09)		15	*−0.36* *(−0.51 to −0.21)*		27	0.02	.35	−0.20 (−0.41 to 0.01)		24	*−0.42* *(−0.65 to −0.19)*		28	0.00	.02^d^
Care recipient need	*−0.18* *(−0.34 to −0.03)*		15	*−0.33* *(−0.56 to −0.11)*		27	0.005	.21	*−0.26* *(−0.43 to −0.09)*		24	*−0.37* *(−0.61 to −0.13)*		28	0.00	.15
Psychological need	*−0.41* *(−0.66 to −0.16)*		15	−0.34 (−0.68 to 0.00)		27	0.02	.61	*−0.44* *(−0.67 to −0.21)*		24	*−0.43* *(−0.73 to −0.13)*		28	0.003	.91
Social support need	*−0.35* *(−0.59 to −0.11)*		15	*−0.38* *(−0.65 to −0.11)*		27	0.00	.72	−0.18 (−0.51 to 0.14)		24	−0.30 (−0.63 to 0.03)		28	0.00	.05
Needs (best practice)	*−0.25* *(−0.36 to −0.15)*		15	*−0.36* *(−0.50 to −0.22*)		27	0.006	.24	*−0.21* *(−0.42 to −0.00)*		24	*−0.36* *(−0.57 to −0.15)*		28	0.00	.004^e^
Resources	*0.14 (0.04 to 0.24)*		15	*0.26 (0.12 to 0.41)*		27	0.007	.10	*0.30 (0.16 to 0.44)*		24	*0.45 (0.32 to 0.58)*		28	0.004	.02^d^
Spirituality	0.01 (−0.12 to 0.15)		15	0.21 (−0.05 to 0.53)		27	0.02	.19	*0.33 (0.15 to 0.51)*		24	*0.46 (0.07 to 0.84)*		28	0.02	.42
Self-efficacy	*0.35 (0.16 to 0.54*)		15	*0.42 (0.11 to 0.73)*		27	0.008	.50	*0.42 (0.12 to 0.73)*		24	*0.49 (0.22 to 0.77)*		28	0.001	.40
Responsibility	0.03 (−0.09 to 0.16)		15	0.08 (−0.23 to 0.38)		27	0.02	.69	0.16 (−0.00 to 0.31)		24	*0.24* *(0.00 to 0.48)*		28	0.006	.41
Community resources	0.07 (−0.63 to 0.76)		16	0.52 (−0.02 to 1.06)		27	0.06	.17	0.41 (−0.25 to 1.07)		26	*0.88 (0.32 to 1.44)*		28	0.11	.23
Family support	*0.19 (0.05 to 0.33)*		15	*0.22 (0.09 to 0.34)*		27	0.00	.59	*0.25 (0.05 to 0.44*)		24	*0.30 (0.17 to 0.42)*		28	0.00	.47
Closeness with care recipient	*0.18* *(0.03 to 0.34)*		15	0.22 (−0.00 to 0.44)		27	0.02	.77	*0.26 (0.03 to 0.48)*		24	*0.46 (0.20 to 0.71)*		28	0.02	.16
Health awareness	*0.21 (0.02 to 0.39)*		15	*0.25 (0.02 to 0.48)*		27	0.00	.47	*0.32 (0.13 to 0.52)*		24	*0.48 (0.29 to 0.68*)		28	0.00	.03^d^
PACS^f^	0.16 (−0.08 to 0.40)		15	0.14 (−0.04 to 0.31)		33	0.01	.77	*0.17 (0.07 to 0.28)*		*24*	*0.26 (0.15 to 0.36)*		34	0.01	.33
Affirming	0.18 ( −0.10 to 0.46)		15	0.06 (−0.15 to 0.28)		33	0.02	.34	*0.20 (0.00 to 0.40)*		24	*0.21 (0.06 to 0.36)*		34	0.02	.95
Enriching	0.15 (−0.06 to 0.35)		15	*0.23 (0.05 to 0.42)*		33	0.00	.23	*0.14 (0.03 to 0.25)*		24	*0.32 (0.16 to 0.47)*		34	0.00	.02^d^
Secondary outcomes																
GHQ-12^g^	*−0.20* *(−0.38 to −0.02)*		15	*−0.13* *(−0.25 to −0.01)*		33	0.02	.35	*−0.20* *(−0.31 to −0.09)*		24	*−0.22* *(−0.31 to −0.14)*		34	0.00	.43
EQ-5D	−0.03 (−0.10 to 0.03)		15	0.01 (−0.06 to 0.08)		33	0.06	.33	0.01 (−0.07 to 0.09)		24	0.03 (−0.03 to 0.09)		34	0.02	.57
Peace of Mind Scale	0.16 (−0.13 to 0.46)		15	0.05 (−0.11 to 0.21)		33	0.03	.38	*0.31 (0.11 to 0.51)*		24	*0.28 (0.12 to 0.43)*		34	0.00	.64

a Imputed data are used. These variables are controlled in the multilevel regression models. Related to caregivers: group allocation, age, sex, education level, married, with full-time job, caregiving stage; related to care recipients: age, sex, activities of daily living, instrumental activities of daily living; relationship between caregivers and recipients using spousal relationship as reference: child and others. Models accounted for clustering at the center level. Intervention effects are reported with bias-reduced linearization (CR2)–adjusted SEs and small-sample corrected degrees of freedom. ICCs quantify the variance attributable to center-level clustering.

b ICC: intraclass correlation.

c Italicized pairs of estimated means and SEs indicate significant within-group differences between T2 (3-month follow-up) and T1 (baseline assessment) or T3 (6-month follow-up) and T1 at 95% CI.

d *P*<.05

e *P*<.01

f PACS: Positive Aspects of Caregiving Scale.

g GHQ-12: 12-item General Health Questionnaire.

### T2-T1 Changes

There were significant within-group changes between 3 months of follow-up (T2) and the baseline (T1). Considering changes within the intervention group, there were significant reductions in overall needs (best practice; 95% CI −0.50 to −0.22), role conflict (95% CI −0.51 to −0.21), care recipient need (95% CI −0.56 to −0.11), social support need (95% CI −0.65 to −0.11), as well as GHQ-12 (95% CI −0.25 to −0.01). There were also significant increases in overall resources (95% CI 0.12‐0.41), specifically self-efficacy (95% CI 0.11‐0.73), family support (95% CI 0.09‐0.34), health awareness (95% CI 0.02‐0.48), and enriching aspect of caregiver (95% CI 0.05‐0.42). In the control group, there were significant decreases in overall needs (best practice; 95% CI −0.36 to −0.15), physiological need (95% CI −0.27 to −0.00), care recipient need (95% CI −0.34 to −0.03), psychological need (95% CI −0.66 to −0.16), social support need (95% CI −0.59 to −0.11), and GHQ-12 (95% CI −0.38 to −0.02), as well as increases in overall resources (95% CI 0.04‐0.24), specifically in self-efficacy (95% CI 0.16‐0.54), family support (95% CI 0.05‐0.33), closeness with care recipient (95% CI 0.03‐0.34), and health awareness (95% CI 0.02‐0.39).

Regarding between-group differences, the multilevel regression revealed no significant intervention effects in T2-T1 changes after adjusting for covariates.

### T3-T1 Changes

Considering the within-group changes between the 6-month follow-up (T3) and the baseline, there were significant decreases in overall needs (best practice; 95% CI −0.57 to −0.15) and 3 subdomains, including role conflict (95% CI −0.65 to −0.19), care recipient need (95% CI −0.61 to −0.13), and psychological need (95% CI −0.73 to −0.13). There were also significant increases in overall resources (95% CI 0.32‐0.58) and all subdomains and positive aspects of caregiving (95% CI 0.15‐0.36) in the intervention group. We also observed significant decreases in care recipient (95% CI −0.43 to −0.09) and psychological needs (95% CI −0.67 to −0.21) in the control group, as well as increases in overall resources (95% CI 0.16‐0.44) and 5 subdomains, including spirituality (95% CI 0.15‐0.51), self-efficacy (95% CI 0.12‐0.73), family support (95% CI 0.05‐0.44), closeness with care recipient (95% CI 0.03‐0.48), and health awareness (95% CI 0.13‐0.52). Both groups exhibited increases in peace of mind (intervention: 95% CI 0.12‐0.43; control: 95% CI 0.11‐0.51) and a decrease in GHQ-12 (intervention: 95% CI −0.31 to −0.14; control: 95% CI −0.31 to −0.09).

Regarding the between-group differences in the change scores between T3 and T1, the improvements of the intervention group were significantly greater than the control group in overall needs (best practice*; b*=−0.15; SE=0.03; *t*_5.96_=−4.54; *P*=.004; ICC<0.001, intervention: 95% CI −0.57 to −0.15 and control: 95% CI −0.42 to −0.00), role conflict (*b*=−0.22; SE=0.07; *t*_5.96_= –3.31; *P*=.02; ICC<0.001, intervention: 95% CI −0.65 to −0.19 and control: 95% CI −0.41 to 0.01), overall resources (*b*=0.15; SE=0.05; *t*_5.99_=2.98; *P*=.02; ICC=0.004, intervention: 95%CI 0.32‐0.58 and control: 95% CI 0.16‐0.44), health awareness (*b*=0.16; SE=0.06; *t*_5.96_=2.85; *P*=.03; ICC<0.001, intervention: 95% CI 0.29‐0.68 and control: 95% CI 0.13 to 0.52), and enriching aspects of caregiving (*b*=0.18; SE=0.06; *t*_5.96_=3.25; *P*=.02; ICC<0.001, intervention: 95% CI 0.16‐0.47 and control: 95% CI 0.03‐0.25). Results of the multilevel regression models are shown in Table 3. The fixed effect full models of significant intervention effects are reported in Table S2 of Multimedia Appendix 1.

### Moderating Factors

We tested moderation effects to examine whether the intervention’s impact differed across caregiver subgroups (Table 4). There were significant negative interactions between the intervention condition and education level (*b*=−0.16, SE=0.06, *t*_550_=−2.80, *P*=.005), as well as between the intervention condition and caregiver of other relations (*b*=−0.45, SE=0.19, *t*_550_=−2.40, *P*=.02) on the T3-T1 needs, indicating a stronger intervention effect in reducing needs among caregivers with higher education levels and in other relationships (nonchild and nonspousal) with the care recipients (as compared to spousal caregivers).

**Table 4.** Multilevel regressions examining moderation effect of demographics on T3 (6-month follow-up) to T1 (baseline assessment) changes in primary outcomes (N=565)^a^.

	Overall needs (best practice)		Overall resources		Affirming		Enriching	
	*b*	*P* value	*b*	*P* value	*b*	*P* value	*b*	*P* value
Intervention	−0.15	.03^b^	0.15	.049^b^	0.01	.94	0.18	.009^c^
Carer								
Age (y)	0.00	.89	0.00	.14	0.00	.41	−0.01	.23
Women	0.03	.76	−0.01	.92	0.05	.69	−0.01	.95
Education	−0.01	.87	−0.05	.06	0.01	.83	−0.03	.28
Marital status	−0.13	.19	0.10	.17	0.02	.85	−0.02	.85
Employment	−0.10	.34	0.11	.18	0.17	.12	−0.01	.93
Caregiving stage	0.05	.49	−0.14	.01^b^	−0.10	.17	−0.19	.006^c^
Care recipient								
Age (y)	0.00	.46	−0.01	.15	−0.01	.07	0.00	.48
Women	0.22	.03^b^	−0.05	.54	−0.01	.91	0.11	.27
ADL^d^	0.01	.48	−0.01	.08	0.00	.74	−0.02	.10
IADL^e^	−0.02	.16	0.00	.95	−0.03	.008^c^	−0.02	.03^b^
Child (reference spouse)	−0.15	.27	0.16	.14	0.14	.33	−0.10	.47
Other relations (reference spouse)	−0.21	.10	0.18	.07	−0.04	.75	−0.14	.25
Moderation^f^								
Carer								
Age	0.01	.32	−0.00	.29	−0.01	.33	−0.00	.64
Women	0.08	.66	0.03	.86	0.03	.88	−0.10	.61
Education	−0.16	.005^c^	−0.02	.64	−0.07	.26	−0.08	.17
Marital status	0.09	.57	0.12	.34	0.22	.18	−0.01	.97
Employment	−0.04	.81	0.18	.17	−0.02	.92	−0.10	.58
Caregiving stage	0.10	.46	0.02	.82	−0.09	.53	0.04	.77
Care recipient								
Age	0.00	.93	0.00	.90	−0.00	.69	−0.01	.48
Women	0.02	.89	−0.08	.46	−0.18	.20	0.02	.88
ADL	−0.03	.07	0.01	.68	0.02	.20	−0.02	.18
IADL	−0.01	.61	0.02	.06	0.02	.34	0.01	.47
Child (reference spouse)	−0.05	.74	−0.00	.99	−0.05	.74	0.01	.92
Other relations (reference spouse)	−0.45	.02^b^	−0.18	.20	0.07	.72	−0.23	.21

a Imputed data are used. Models accounted for clustering at the center level. Intervention effects are reported with bias-reduced linearization (CR2)–adjusted SEs and small sample–corrected *df*.

b *P*<.05.

c *P*<.01.

d ADL: activities of daily living.

e IADL: instrumental activities of daily living.

f Only the coefficients relevant to the moderators in the multilevel regressions are shown.

## Discussion

### Principal Findings

This study demonstrates the effectiveness of the CSM in reducing caregiver burden and enhancing resources among informal caregivers of frail older adults. The predominantly female caregiver population, many managing their own chronic health conditions, underscores the dual challenges these individuals face [35]. Balancing personal health with caregiving responsibilities highlights the necessity for integrated support systems that address both the demands of caregiving and the caregivers’ personal well-being.

### Intervention Effectiveness

The CSM intervention yielded more pronounced effects at 6-month (T3) follow-ups compared to 3-month (T2) follow-ups, indicating sustained and consolidating benefits over time. Caregivers reported significant improvements in overall needs, suggesting a reduced sense of burden in managing caregiving responsibilities. The reduction in role conflict suggests that caregivers became more adept at balancing caregiving tasks with personal obligations, likely supported by the CSM’s structured approach, which offers practical strategies for prioritizing duties and handling competing demands. Personalized intervention plans, guided by the CNRA subscores, enabled targeted support and resource allocation to meet the needs and circumstances of each caregiver, thereby enhancing their ability to address both the practical and emotional needs of frail family members.

The intervention also led to significant increases in overall resources, especially in health awareness. This suggests that the CSM supported caregivers in recognizing and prioritizing their own health needs, which in turn enhances their ability to sustain caregiving over time. By strengthening such personal resources, the intervention contributed to building a more resilient foundation for caregivers’ well-being alongside their caregiving responsibilities.

Caregivers also experienced significant enhancements in the enriching aspects of caregiving, reporting greater fulfillment and meaning in their roles. This positive shift in perspective suggests that the intervention helped them recognize the personal growth and skills gained from their caregiving responsibilities. In addition, acknowledgment of their caregiving efforts, both from the intervention team and their wider support network, may also have contributed to these feelings of enrichment.

These findings are consistent with the stress process model [11], which emphasizes the importance of reducing secondary strains such as role conflict, and of strengthening mediating resources. In our results, the intervention reduced role conflict and enhanced caregivers’ sense of health awareness, suggesting improved capacity for coping and self-regulation in the caregiving role. The sustained benefits at 6 months are consistent with the stress and coping model [12,13], in which repeated cycles of appraisal (baseline assessment), coping (individualized service planning), and reappraisal (follow-up reviews) enable caregivers to adapt more effectively over time. They also resonate with strengths-based frameworks [14], which emphasize bolstering caregivers’ self-efficacy and resourcefulness to manage demands more effectively. By lowering perceived burdens and expanding support systems, the CSM fostered resilience and enhanced caregivers’ well-being.

Importantly, the CSM is designed as a personalized intervention in which social workers adjust the content and focus of support according to each caregiver’s CNRA scores. This individualized tailoring means that the program emphasizes strengthening caregivers’ overall capacity by addressing their most salient and immediate needs, rather than targeting all domains uniformly. Consequently, it is reasonable that the most consistent improvements were observed in overall needs and overall resources, while effects on specific sub-domains varied. This pattern reflects the program’s emphasis on adaptive, needs-driven support rather than standardized delivery across all participants.

### Moderating Factors and Tailored Interventions

The varying effectiveness of the intervention across different caregiver subgroups highlights the influence of factors such as the caregiver’s relationship with the care recipient and education level. Caregivers in other relationships and those with higher education levels experienced more significant benefits from the intervention than the spousal caregivers. Nonspousal caregivers may face fewer emotional complexities, allowing them to engage more readily with practical caregiving strategies provided by the CSM. Conversely, spousal caregivers might experience greater emotional strain when caring for a partner, which can affect their ability to fully use the intervention’s resources [36]. Caregivers with higher education levels might find it easier to comprehend and implement the intervention components because of better access to information and learning resources [37]. These findings suggest the need for tailored interventions to support spousal caregivers and those with lower education levels more effectively. Spousal caregivers might benefit from additional emotional support and relational counseling to address the unique challenges they face [38]. Simplified educational materials and training programs could enhance accessibility for caregivers with lower educational backgrounds, ensuring they can fully engage with the intervention. Reflecting ongoing input from frontline practitioners and service users, some refinements have also been made to the wording of CNRA items to improve clarity and accessibility (ie, the 3-item closeness with the care recipient subscale; Leung et al, unpublished data), particularly for caregivers with lower literacy levels.

### Practical Implications

The study’s findings have significant practical implications for the design and implementation of caregiver support programs. Interventions should be customized to address the specific challenges faced by diverse caregiver groups, incorporating stress management techniques, health monitoring, and financial planning assistance to alleviate the compounded burdens of caregiving and personal health management [39]. Developing educational resources tailored to varying literacy levels can empower all caregivers with the necessary knowledge and skills, enhancing the overall effectiveness of support programs [40]. The need for sustained improvement among caregivers highlights the importance of long-term, adaptive interventions. Continuous training, regular reassessment of needs, and personalized care plans can help maintain and enhance the benefits of the intervention over time.

Moreover, the findings underscore the importance of policy initiatives and community involvement in supporting caregivers. Policymakers should consider allocating resources to develop and maintain evidence-based caregiver support programs like the CSM, recognizing the critical role caregivers play in the health system. Community engagement is essential for raising awareness about the challenges caregivers face and mobilizing support networks, which can provide additional layers of assistance and relief [41].

### Limitations and Future Research

Despite the positive outcomes, the study has several limitations that should be considered. First, the study was conducted during the COVID-19 pandemic, which necessitated adaptations in service delivery, shifting from face-to-face interactions to virtual platforms, which may have influenced the outcomes and limited the generalizability of the findings to nonpandemic contexts. Future research should examine the long-term effectiveness of the CSM in an after-pandemic context to validate these findings. Second, the sample predominantly consisted of female caregivers recruited from 8 centers associated with 4 NGOs in Hong Kong, which may limit the applicability of the findings to male caregivers or those in different regions. Third, the reliance on self-reported measures introduces the possibility of response biases, such as social desirability or recall bias. Fourth, the follow-up period was limited to 6 months, leaving the long-term durability of the intervention’s effects uncertain. Future research should extend follow-up periods and incorporate objective measures to validate and expand upon these findings. Fifth, although moderation effects were examined using interaction terms in separate multilevel models, our sample size (N=565) may not have been sufficient to detect small interaction effects, which often require much larger samples (eg, 800‐1000) to achieve adequate power [42]. Nonsignificant moderation findings should therefore be interpreted with caution. Sixth, while the intervention produced statistically significant improvements, it remains unclear whether these changes meet clinical or practical thresholds. Minimal clinically important differences for most caregiver-reported outcomes, except GHQ-12, have not been established. Referencing the 11/12 threshold used in a local study using the Likert scoring method [43], around 73% (205/281) of caregivers in the intervention group exceeded this cutoff at baseline, indicating high distress levels in the caregiving role, while the percentage fell to 64% (180/281) at T3, suggesting potential benefits of the intervention in alleviating psychological distress, although the difference in the change of GHQ-12 scores between the intervention and control groups is not significant. Future research should define minimal clinically important differences for other key outcomes to improve the interpretation of intervention effects [1]. Finally, future research should also evaluate whether the CSM can reduce frail older adults’ health care use, such as hospital admissions or emergency visits, and overall system costs, thereby demonstrating their broader value to health and social care systems.

### Conclusions

The CSM offers an effective, structured approach for reducing caregiver burden and enhancing resources among informal caregivers of frail older adults. By offering personalized support and practical tools, the CSM addresses the multifaceted challenges caregivers face. The findings of this study highlight the necessity for long-term adaptable interventions that can evolve with caregivers’ changing needs. Implementing such models can significantly improve caregivers’ well-being and contribute to a more resilient caregiving system, which is increasingly important in the context of an aging global population.

## Supplementary material

10.2196/71638 Multimedia Appendix 1 Detailed intervention protocol of caregiver support model and supplementary results.

10.2196/71638 Checklist 1 CONSORT-eHEALTH checklist.

## Abbreviations

CNRA: Caregiver Needs and Resources Assessment
CONSORT-EHEALTH: Consolidated Standards of Reporting Trials of Electronic and Mobile Health Applications and Online Telehealth
CSM: caregiver support model
GHQ-12: 12-item General Health Questionnaire
ICC: intraclass correlation coefficient
NGO: nongovernmental organization
PACS: Positive Aspects of Caregiving Scale
PoMS: Peace of Mind Scale
ZBI-12: 12-item Zarit Burden Interview
