# Supplementary material for: The Caregiver Support Model for Informal Caregivers of Frail Older Adults: Randomized Controlled Trial
Source: JMIR Aging. 2025 Nov 3;8:e71638. doi: 10.2196/71638 (PMC12582877; doi:10.2196/71638)
Supplement: Multimedia Appendix 1 [file aging-v8-e71638-s001.docx]

**APPENDIX**

**Appendix 1: Detailed Intervention Protocol of CSM**

1. Initial Assessment with CNRA:

- Eligible caregivers completed the Caregiver Needs and Resources Assessment (CNRA), which evaluates needs and resources across multiple dimensions.
- Social workers in the intervention group reviewed CNRA scores before the first meeting with caregivers.
- In the control group, social workers did not have access to CNRA scores and based interventions on personal judgment.

1. First Meeting:

- Conducted within one week of CNRA completion.
- Social workers discussed CNRA results with caregivers, focusing on prioritizing needs and proposing initial intervention plans.
- Peer mentors were randomly assigned to caregivers (one mentor for every five participants).
- If the first meeting could not occur within six weeks after the baseline CNRA, the CNRA had to be redone.

1. Adaptations made during the COVID-19 Pandemic:

- Flexibility in meeting formats (face-to-face, video calls, Zoom meetings) due to the pandemic.
- Relaxed timeline for initial meetings during the outbreak to six weeks.

1. Second Meeting:

- Held one week after the first meeting.
- Reviewed any changes in caregivers' situations and discussed strategies to utilize their resources.

1. Regular Follow-ups and Midterm Evaluation:

- The social workers were expected to meet with the caregiver participant for follow-up every month during the outbreak, with each meeting no less than 15 minutes.
- Midterm evaluation at three months involved reviewing the second CNRA scores and adjusting intervention plans.
- At three months, a midterm evaluation was done with the caregiver, reviewing the second CNRA scores and comparing them with the first CNRA results.
- Depending on the caregiver's situation, social workers might adjust existing intervention plans.
- At four and five months, the social worker met with the caregiver to monitor treatment progress.

1. Final Assessment and Termination:

- The intervention concluded with a final meeting after the six-month CNRA.
- Overall progress was evaluated, and decisions were made regarding the continuation or termination of the intervention.
- T3 CNRA was conducted six months after intake, and the scores were uploaded to the online caregiver management system.
- The last meeting with the caregiver was held after receiving the T3 CNRA scores to decide on the future course of the intervention.

1. Control Group Protocol:

- Social workers managed case interventions independently, without CNRA scores.
- A similar schedule for assessments as the intervention group.

##

| 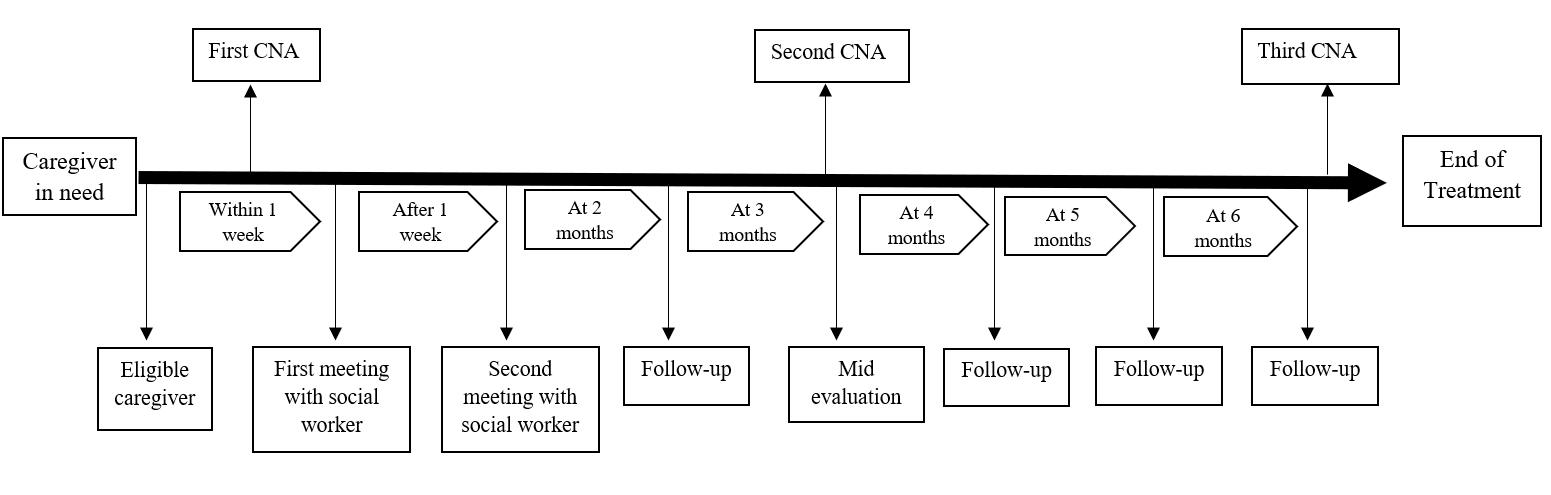 |
| --- |
| **Figure S1.** Intervention Flow |

| **Form 2** **“Caregiver Support Model” Project: Caregiver Needs Assessment**  **Case Number: ______**  Dear Caregiver,  The “Caregiver Support Model” project is organized by City University of Hong Kong with support from the Simon K.Y. Lee Elderly Fund. Its aim is to understand the difficulties and needs of family caregivers when looking after older adults, as well as the physical and psychological changes of caregivers themselves. Using data analysis, the study hopes to explore ways to improve caregiver support services. The study runs from November 2021 to December 2022.  This questionnaire survey is mainly designed to understand the physical and psychological changes of family caregivers during the caregiving process, and the activities they participate in during caregiving. Each caregiver will be surveyed three times, each session lasting about 30 minutes. The first survey will be conducted shortly after you agree to participate; the second survey will be three months later; and the third survey will be six months after that. The information you provide will only be used for academic research purposes. Participation is entirely voluntary, and you may withdraw at any time. For the first, second, and third surveys, we will each provide supermarket coupons valued at HK$50, HK$100, and HK$150 respectively, as a token of appreciation.  If you have any questions regarding the survey, please feel free to contact our research assistant (Tel: XXXX / Email: [XXXX@cityu.edu.hk](mailto:XXXX@cityu.edu.hk)).  ______________________________________________________________________  **Research Participation Consent Form**   1. I am currently a family caregiver. 2. I have read and understood the purpose of this survey and the related information, and I agree to participate voluntarily. 3. I understand that all personal data and information provided will be kept strictly confidential, and all research data will be destroyed one year after the project is completed.   Signature: ___________________________  Name: Mr./Ms. __________________  Date: __________________ |
| --- |

| **Form 5 Consent to Receive Services and Provide Personal Information**  I agree to receive the services provided to me by this center, and I fully understand and consent to the following contents:   - The personal information you provide will be recorded in the case file and used for future service planning, statistical reporting, and provision of service information, such as program applications and activity arrangements. - Your personal information may be randomly selected for review by funding organizations for service monitoring purposes. - If you later object to the continued use of your personal information for the above-mentioned purposes, please notify the center in writing, and your personal information will be deleted to facilitate processing. - To protect your privacy, all collected information will be securely handled and kept confidential. It will not be sold, rented, or disclosed in any form to any individual or organization. All information will be destroyed one year after the completion of services, in accordance with policy regulations. - You may request to correct, review, or obtain your personal information, but a written application is required. We will respond and process within 40 working days. - If you wish to withdraw from services, please notify the responsible social worker.   **Service User’s Name:** ___________ **Service User’s Signature:** ___________ **Date:** ___________  **Responsible Social Worker’s Name:** ___________ **Responsible Social Worker’s Signature:** ___________ **Date:** ___________ |
| --- |
| **Figure S2.** Informed Consent Form (translated into English) |

|  |
| --- |
|  |
| **Figure S3.** Sample spider web charts of needs and resources of the caregiver |

|  |
| --- |

| **Table S1. Descriptive statistics of the participants across participating centers (N = 565)** | | | | | | | | | | |
| --- | --- | --- | --- | --- | --- | --- | --- | --- | --- | --- |
| **Characteristics of the caregivers** | **Center 1** n = 68*^1^* | **Center 2** n = 71*^1^* | **Center 3** n = 73*^1^* | **Center 4** n = 76*^1^* | **Center 5** n = 72*^1^* | **Center 6** n = 71*^1^* | **Center 7** n = 72*^1^* | **Center 8** n = 62*^1^* | ***P****^2^* | |
| Age | 68 (9) | 64 (14) | 70 (10) | 63 (13) | 71 (9) | 63 (14) | 68 (9) | 69 (11) | <0.001 | ******* |
| Women | 60 (88%) | 61 (86%) | 59 (81%) | 63 (83%) | 59 (82%) | 65 (92%) | 61 (85%) | 50 (81%) | .60 |  |
| Married | 48 (71%) | 48 (68%) | 55 (75%) | 60 (79%) | 62 (86%) | 47 (66%) | 61 (85%) | 43 (69%) | .03 |  |
| **Characteristics of the care recipients** |  |  |  |  |  |  |  |  |  |  |
| Age | 81 (9) | 81 (10) | 83 (10) | 80 (9) | 81 (7) | 82 (8) | 80 (9) | 82 (10) | .50 |  |
| Women | 26 (38%) | 31 (44%) | 43 (59%) | 34 (45%) | 21 (29%) | 28 (39%) | 25 (35%) | 24 (39%) | .02 | ***** |
| IADL | 4.5 (2.8) | 9.0 (3.9) | 8.9 (3.8) | 9.6 (5.1) | 9.9 (4.0) | 8.6 (3.7) | 7.2 (3.6) | 6.5 (5.1) | <0.001 | ******* |
| ADL | 2.0 (2.8) | 5.0 (4.1) | 4.8 (3.9) | 7.2 (4.2) | 4.1 (4.5) | 3.3 (4.3) | 2.3 (4.1) | 3.4 (3.3) | <0.001 | *** |
| *^1^* n (%); Mean (SD)  *^2^* Pearson’s Chi-squared test; One-way analysis of means  *** *P* < .001; ** *P* < .01; * *P* < .05. | | | | | | | | | | |

| **Table S2. Multilevel regressions examining moderation effect of demographics on T3-T1 changes in primary outcomes (*N* = 565)** | | | | | | |
| --- | --- | --- | --- | --- | --- | --- |
| DV: Overall Need (Best Practice) | *b* | SE | df | *t* | *P* |  |
| Intervention | -0.15 | 0.07 | 551 | -2.12 | .03 | * |
| Carer |  |  |  |  |  |  |
| Age | 0.00 | 0.00 | 551 | 0.14 | .89 |  |
| Women | 0.03 | 0.12 | 551 | 0.30 | .76 |  |
| Education | -0.01 | 0.03 | 551 | -0.16 | .87 |  |
| Marital status | -0.13 | 0.10 | 551 | -1.32 | .19 |  |
| Employment | -0.10 | 0.11 | 551 | -0.95 | .34 |  |
| Caregiving stage | 0.05 | 0.07 | 551 | 0.70 | .49 |  |
| Care recipient |  |  |  |  |  |  |
| Age | 0.00 | 0.00 | 551 | -0.74 | .46 |  |
| Women | 0.22 | 0.10 | 551 | 2.12 | .03 | * |
| ADL | 0.01 | 0.01 | 551 | 0.70 | .48 |  |
| IADL | -0.02 | 0.01 | 551 | -1.42 | .16 |  |
| Child (ref: spouse) | -0.15 | 0.14 | 551 | -1.10 | .27 |  |
| Other relations (ref: spouse) | -0.21 | 0.13 | 551 | -1.63 | .10 |  |
| DV: Role Conflict | *b* | SE | df | *t* | *P* |  |
| Intervention | -0.22 | 0.09 | 551 | -2.47 | .01 | * |
| Carer |  |  |  |  |  |  |
| Age | 0.00 | 0.01 | 551 | -0.24 | .81 |  |
| Women | 0.08 | 0.15 | 551 | 0.55 | .58 |  |
| Education | -0.02 | 0.04 | 551 | -0.52 | .60 |  |
| Marital status | -0.03 | 0.13 | 551 | -0.28 | .78 |  |
| Employment | -0.20 | 0.14 | 551 | -1.45 | .15 |  |
| Caregiving stage | 0.04 | 0.09 | 551 | 0.40 | .69 |  |
| Care recipient |  |  |  |  |  |  |
| Age | -0.01 | 0.01 | 551 | -1.90 | .06 |  |
| Women | 0.17 | 0.13 | 551 | 1.24 | .21 |  |
| ADL | 0.00 | 0.01 | 551 | 0.35 | .73 |  |
| IADL | 0.00 | 0.01 | 551 | -0.17 | .87 |  |
| Child (ref: spouse) | -0.02 | 0.18 | 551 | -0.12 | .91 |  |
| Other relations (ref: spouse) | -0.18 | 0.16 | 551 | -1.10 | .27 |  |
| ***Notes.*** Imputed data is used. Models accounted for clustering at the center level. Intervention effects are reported with CR2-adjusted standard errors and small-sample corrected degrees of freedom.  Degrees of freedom (df) were calculated using Satterthwaite approximations. For outcomes where cluster-level variance contributed to the model, dfs reflect the effective number of clusters rather than the total sample size.  *** *P* < .001; ** *P* < .01; * *P* < .05. | | | | | | |

| **Table S2. Multilevel regressions examining moderation effect of demographics on T3-T1 changes in primary outcomes (*N* = 565)** | | | | | | |
| --- | --- | --- | --- | --- | --- | --- |
| DV: Overall Resource | *b* | SE | df | *t* | *P* |  |
| Intervention | 0.15 | 0.06 | 5.37 | 2.53 | .049 | * |
| Carer |  |  |  |  |  |  |
| Age | 0.00 | 0.00 | 350.40 | 1.48 | .14 |  |
| Women | -0.01 | 0.09 | 549.78 | -0.10 | .92 |  |
| Education | -0.05 | 0.02 | 452.07 | -1.91 | .06 |  |
| Marital status | 0.10 | 0.07 | 549.36 | 1.36 | .17 |  |
| Employment | 0.11 | 0.08 | 549.34 | 1.33 | .18 |  |
| Caregiving stage | -0.14 | 0.05 | 545.03 | -2.57 | .01 | * |
| Care recipient |  |  |  |  |  |  |
| Age | -0.01 | 0.00 | 549.03 | -1.46 | .15 |  |
| Women | -0.05 | 0.08 | 530.67 | -0.62 | .54 |  |
| ADL | -0.01 | 0.01 | 355.97 | -1.77 | .08 |  |
| IADL | 0.00 | 0.01 | 285.53 | 0.07 | .95 |  |
| Child (ref: spouse) | 0.16 | 0.11 | 503.81 | 1.49 | .14 |  |
| Other relations (ref: spouse) | 0.18 | 0.10 | 525.96 | 1.83 | .07 |  |
| DV: Health Awareness | *b* | SE | df | *t* | *P* |  |
| Intervention | 0.16 | 0.09 | 551 | 1.73 | .08 |  |
| Carer |  |  |  |  |  |  |
| Age | 0.02 | 0.01 | 551 | 2.96 | .003 | ** |
| Women | 0.08 | 0.15 | 551 | 0.51 | .61 |  |
| Education | -0.02 | 0.04 | 551 | -0.44 | .66 |  |
| Marital status | 0.04 | 0.13 | 551 | 0.31 | .76 |  |
| Employment | 0.09 | 0.14 | 551 | 0.65 | .52 |  |
| Caregiving stage | -0.15 | 0.09 | 551 | -1.61 | .11 |  |
| Care recipient |  |  |  |  |  |  |
| Age | -0.01 | 0.01 | 551 | -1.22 | .22 |  |
| Women | -0.22 | 0.13 | 551 | -1.64 | .10 |  |
| ADL | -0.01 | 0.01 | 551 | -0.90 | .37 |  |
| IADL | 0.02 | 0.01 | 551 | 1.76 | .08 |  |
| Child (ref: spouse) | 0.14 | 0.18 | 551 | 0.75 | .45 |  |
| Other relations (ref: spouse) | 0.27 | 0.16 | 551 | 1.62 | .11 |  |
| DV: Enrich Aspects of Caregiving | *b* | SE | df | *t* | *P* |  |
| Intervention | 0.18 | 0.07 | 551 | 2.62 | .009 | ** |
| Carer |  |  |  |  |  |  |
| Age | -0.01 | 0.00 | 551 | -1.20 | .23 |  |
| Women | -0.01 | 0.11 | 551 | -0.06 | .95 |  |
| Education | -0.03 | 0.03 | 551 | -1.08 | .28 |  |
| Marital status | -0.02 | 0.10 | 551 | -0.19 | .85 |  |
| Employment | -0.01 | 0.11 | 551 | -0.09 | .93 |  |
| Caregiving stage | -0.19 | 0.07 | 551 | -2.74 | .01 | ** |
| Care recipient |  |  |  |  |  |  |
| Age | 0.00 | 0.00 | 551 | -0.71 | .48 |  |
| Women | 0.11 | 0.10 | 551 | 1.11 | .27 |  |
| ADL | -0.02 | 0.01 | 551 | -1.66 | .10 |  |
| IADL | -0.02 | 0.01 | 551 | -2.13 | .03 | * |
| Child (ref: spouse) | -0.10 | 0.14 | 551 | -0.73 | .47 |  |
| Other relations (ref: spouse) | -0.14 | 0.13 | 551 | -1.15 | .25 |  |
| ***Notes.*** Imputed data is used. Models accounted for clustering at the center level. Intervention effects are reported with CR2-adjusted standard errors and small-sample corrected degrees of freedom.  Degrees of freedom (df) were calculated using Satterthwaite approximations. For outcomes where cluster-level variance contributed to the model, dfs reflect the effective number of clusters rather than the total sample size.  *** *P* < .001; ** *P* < .01; * *P* < .05. | | | | | | |
|  |  |  |  |  |  |  |
